# A Case of a Hemodialysis Patient With Myelodysplastic Neoplasm-Like Anemia Due to Hypozincemia Treatment

**DOI:** 10.7759/cureus.79602

**Published:** 2025-02-24

**Authors:** Masanori Kato, Takafumi Fujita, Kiryu Yoshida, Manabu Matsunawa, Hidetoshi Ito

**Affiliations:** 1 Division of Nephrology, Department of Internal Medicine, Showa University Northern Yokohama Hospital, Yokohama, JPN; 2 Division of Hematology, Department of Internal Medicine, Showa University Northern Yokohama Hospital, Yokohama, JPN

**Keywords:** hemodialysis, hypocopperemia, hypozincemia, polaprezinc, zinc acetate

## Abstract

A 50-year-old woman had been on maintenance hemodialysis and used an erythropoiesis-stimulating agent for renal anemia. She developed anemia of unknown cause, and hypozincemia was observed; zinc acetate hydrate was started. However, the improvement was temporary. Leukopenia and macrocytic anemia were observed since the same period, and bone marrow examination revealed abnormal blood cell morphology resembling that of a myelodysplastic neoplasm. She also showed significant hypocopperemia, and we suspected a hematopoietic disorder due to copper deficiency. Zinc acetate hydrate was discontinued, and copper replacement therapy was started. After three weeks, anemia and hypocopperemia improved. Zinc supplementation is sometimes given to hemodialysis patients, but it is important to measure blood copper regularly as zinc supplementation can cause hypocopperemia.

## Introduction

In hemodialysis patients, the progression of anemia is assumed to be caused by various factors such as renal anemia associated with decreased erythropoietin production, suppression of erythropoiesis by uremic toxins, shortened erythrocyte life span, impaired iron metabolism, residual blood and bleeding due to the dialysis circuit, and nutritional disorders.

Adult hemodialysis patients often receive erythropoiesis-stimulating agents (ESAs) and iron supplements for iron deficiency to achieve Hb 10-12 g/dL, but it is estimated that approximately 10% of patients have a poor response to ESA [1].

Zinc deficiency is a contributing factor, and it has also been reported that changes in serum zinc concentrations are independent factors that significantly influence ESA responsiveness in hemodialysis patients [2].

Therefore, supplementation therapy with polaprezinc and zinc acetate, among others, is sometimes used for hypozincemia; however, since zinc inhibits copper absorption in the intestinal tract, hypocopperemia has been reported to occur with the use of zinc preparations.

In this report, we describe a case of anemia in a patient undergoing maintenance hemodialysis who developed hypocopperemia while taking zinc acetate and showed bone marrow findings similar to myelodysplastic neoplasm.

## Case presentation

The case is a woman in her 50s. She visited another hospital complaining of dizziness and lightheadedness. She had a medical history of alcoholism and cerebral hemorrhage in her 40s. Her family history includes liver cirrhosis in her father and type 2 diabetes in her mother. She was a non-smoker and drank 300 ml of shochu (Japanese liquor similar to vodka) twice a week. She was taking precipitated calcium carbonate 1000 mg, folic acid 5 mg, daprodustat 10 mg, zinc acetate hydrate 50 mg, lansoprazole 30 mg, nalfurafine hydrochloride 2.5 μg, and warfarin potassium 1 mg as a regular medication.

She visited our hospital eight years ago for chronic kidney disease (CKD) caused by nephrosclerosis. Seven years ago, she was found to have normocytic normochromic anemia with no increase in reticulocytes and only a mildly elevated erythropoietin level of 54.8 mIU/mL and was started on darbepoetin alfa with the diagnosis of renal anemia.

Six years ago, she had been undergoing hemodialysis because of end-stage renal failure. After being shifted to maintenance dialysis three times a week, she regularly received darbepoetin alfa. However, her anemia progressed and her Hb count was 7.5 g/dL under 60 μg/week of darbepoetin alfa and 40 mg/week of saccharated ferric oxide.

The examination revealed macrocytic anemia with an MCV of 103 fl, but no vitamin B12, folate, or iron deficiency (iron (Fe) 193 μg/dL, total iron-binding capacity (TIBC) 399 μg/dL, transferrin saturation (TSAT) 48.4%, and ferritin (Fer) 47.6 ng/mL).

The upper endoscopy performed to investigate the cause of the anemia showed no evidence of bleeding and fecal occult blood was negative.

As the possibility of hematological disease could not be ruled out, a bone marrow examination was also performed. Pathological findings included normal bone marrow tissue with no anemia-related or chromosomal abnormalities.

Despite symptomatic treatment with darbepoetin alfa at a gradually increasing dose of 180 μg/week, the patient remained anemic, with Hb < 7.0 g/dL, and required a transfusion of four units of red cell concentrates every three weeks.

Anti-erythropoietin antibody was also negative, and the patient did not respond to a change to epoetin beta-pegol 250 μg bi-weekly; therefore, the bone marrow examination was repeated at another hospital approximately four years ago.

Bone marrow findings were a mixture of hypocellular and hypercellular bone marrow, but all stages of maturation were maintained, and no hematological disorders were diagnosed. The patient was changed to epoetin beta-pegol 250 μg every second week; after approximately three months, Hb 11.7 g/dL was achieved, and the patient was followed up.

One year ago, she presented ESA hyporesponsive anemia with hemoglobin (Hb) 8-9 g/dL, TSAT 50.7%, and ferritin 599 ng/mL; folate and zinc deficiencies were suspected. The folic acid concentration in the blood was 0.9 ng/mL, and the zinc concentration was low at 55 μg/dL.

The anemia improved to Hb 10.2-10.7 g/dL, but six months ago, the anemia progressed again to Hb 7.6 g/dL. The patient's Hb count remained below 8.0 g/dL despite a change from epoetin beta-pegol 250 μg/every second week to daprodustat 4-10 mg/week with a gradual increase.

**Figure 1:**
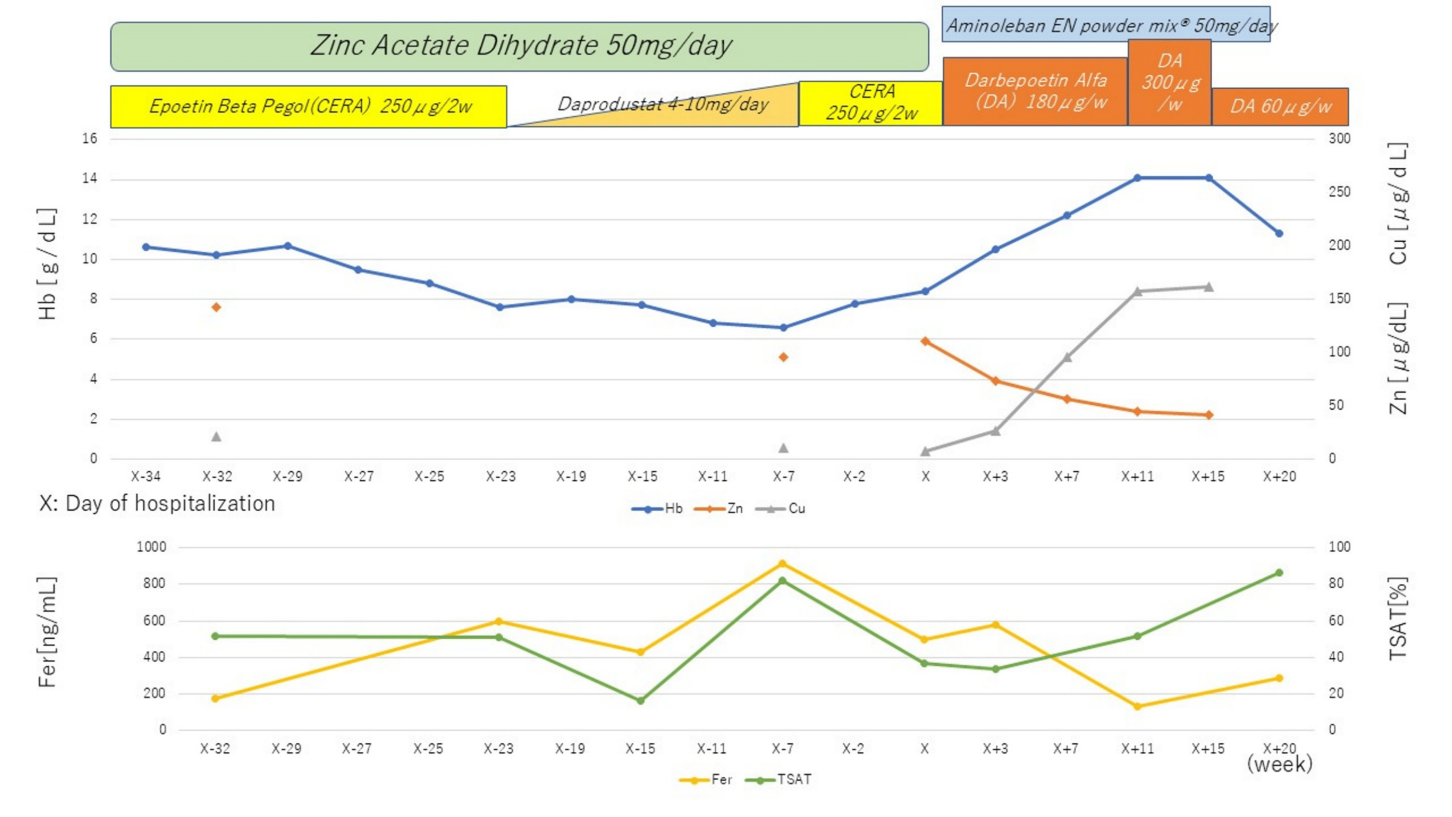
Course of the anemia treatment Hb: hemoglobin, Zn: zinc, Cu: copper, Fer: ferritin, TSAT: transferrin saturation

She was suspected of having gastrointestinal bleeding and underwent an upper endoscopy, but there were no significant findings. She was repeatedly transfused two units of red cell concentrates every four weeks and had iron overload (TSAT 80.1%, Fer 846 ng/mL). She was admitted for further evaluation and treatment (Figure 1).

Post-hospitalization progress

Physical findings upon admission revealed clear consciousness and no disorientation, with a height of 159 cm, weight of 64.7 kg, BMI of 25.6, pulse of 89/min (steady), and blood pressure of 100/56 mmHg. The conjunctiva showed signs of anemia, with no yellowing and no lymphadenopathy. In the chest, no heart murmur was observed, with clear breathing. The abdomen was flat, soft, and tender. The extremities had no skin rash or edema of the lower legs.

On admission, electrocardiogram results were sinus rhythm, pulse of 86/min, and no ST segment changes. Chest X-ray results were a cardiothoracic ratio of 51%, with no lung field abnormalities or pleural effusion.

In this case, leukocytes were 2740/μL, Hb was 8.4 g/dL, MCV was 108 fL, and Plt was 139,000/μL. The patient had pancytopenia with decreased leukocytes and red blood cells. Platelet counts were slightly below the normal lower limit.

Diseases that cause pancytopenia include vitamin B12 deficiency, folic acid deficiency, systemic lupus erythematosus (SLE), liver cirrhosis, and myelodysplastic neoplasm. Vitamin B12 was 460 pg/mL (reference 233-914 pg/mL) and folic acid was 12.8 ng/mL (reference 3.6- 12.9 ng/mL), showing no decrease.

She did not meet the diagnostic criteria for SLE other than leukopenia. She had a history of heavy alcohol consumption, and abdominal ultrasonography performed six months ago showed liver surface irregularities, a spleen index of 23, mild splenomegaly, and elevated fibrosis markers such as hyaluronic acid, collagen type IV7S, and M2BPGi, which were consistent with the diagnosis of cirrhosis but lacked thrombocytopenia, considered atypical for anemia due to cirrhosis.

**Figure 2:**
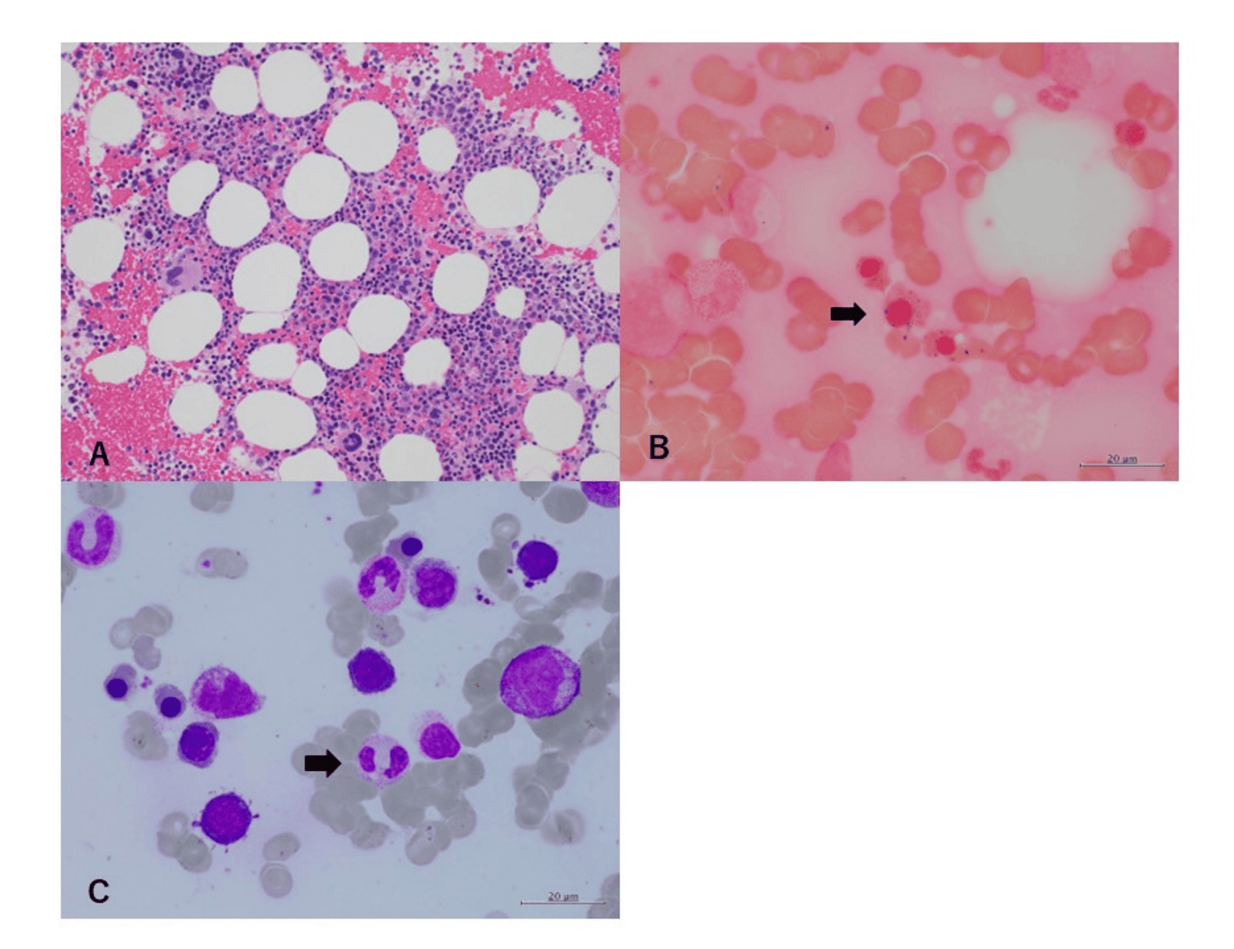
Bone marrow examination A: Normocelluar marrow, H-E stain (×40). B: Ring sideroblast, iron stain (×1000). C: Pseudo Pelger-Huet anomaly, Wright-Giemsa stain (×1000).

The bone marrow examination revealed that the hematopoietic was a normocellular marrow with a cell density of approximately 30-40%. The percentage of blasts was 1.4%, which did not increase, but there were some myelodysplastic syndrome-like blood cell morphological abnormalities, such as annular sideroblasts and hypofractionated neutrophils (Pseudo Pelger-Huet anomaly) (Figure 2).

In addition, since the patient had been taking zinc preparations, the clinic had been conducting periodic zinc testing, and the serum zinc level was 96 μg/dL approximately one month before admission.

Copper, whose absorption is supposed to be antagonistic to that of zinc, as indicated in the package insert, was 11 μg/dL. Examination at the time of admission to our hospital also showed marked hypocupremia, with 111 g/dL of zinc and 8 g/dL of copper (references 66-130 μg/dL). Since copper deficiency is reported to cause blood cell abnormalities similar to myelodysplastic syndrome, we suspected hematopoietic disorder due to hypocupremia.

Zinc acetate hydrate was discontinued, cocoa (6 g pure cocoa contains 0.24 mg copper) was encouraged for copper supplementation, and 50 g/day of Aminoleban EN Combination Acid® (containing 0.2 mg copper) was started. After three weeks, Hb and Cu improved to 10.5 g/dL and 27 μg/dL, respectively, and after seven weeks, Hb and Cu reached normal levels of 12.2 g/dL and 96 μg/dL, respectively.

The patient’s Hb level continued to increase to 14 g/dL, which made it possible to reduce the dose of darbepoetin-alpha to 15 μg/week (Figure 1).

## Discussion

An adult body contains roughly 100 mg of copper, of which about 65% is distributed in the muscles and bones and approximately 10% in the liver [3], and is believed to be involved in energy generation, iron metabolism, maturation of extracellular matrix, production of neurotransmitters, and removal of reactive oxygen species [4].

Dietary copper is taken up by cells by binding to copper transporter 1 (CTR 1) in the mucosal epithelium of the small intestine and is then excreted into the portal vein by ATPase 7A on the basement membrane and into the liver, where it is released as ceruloplasmin into the blood.

Patients with copper deficiency have been reported to receive enteral or intravenous nutritional supplements with low copper content for more than four weeks, which can be caused by resection of the upper part of the small intestine where copper is absorbed, atrophy of the villi of the small intestinal mucosa due to the absence of the intestinal tract, and insufficient nutrients supplied to the upper part of the small intestine due to gastrointestinal bypass surgery.

A metal-binding protein called metallothionein is thought to be responsible for the mechanism of copper deficiency caused by excess zinc, which reduces metal toxicity and maintains homeostasis in small intestinal cells and is induced by zinc stimulation [5], and since metallothionein has a higher affinity for copper than for zinc, it becomes a binding protein and is excreted in the intestinal mucosa and feces [6].

Symptoms of copper deficiency include spinal cord disorders such as gait disturbance, blood cell abnormalities, and hematopoietic disorders [7-9], and as for blood cell abnormalities, anemia, and leukopenia are common, and thrombocytopenia is rare [10].

Bone marrow findings include the appearance of erythroblastoid cells with numerous small vacuoles, hyperplasia of erythroblastoid cells and erythropoiesis, the appearance and leftward migration of numerous small vacuoles in myelomonocytic cells, and ring sideroblast, which should also be differentiated from myelodysplastic syndrome [11,12].

In addition to leukopenia and anemia, a hematopoietic disorder with annular sideroblasts resembling myelodysplastic syndrome was observed in this case; however, no spinal cord injury was observed.

In general, nutritional disorders and trace element deficiencies are expected in dialysis patients due to dietary restrictions and comorbidities; however, serum copper is slightly elevated [13], and hypocopperemia does not seem to occur unless there are causes such as those mentioned above.

On the other hand, zinc is often deficient, and according to a review of 518 dialysis patients by Nagano et al. [14], 264 (51%) were reported to have zinc deficiency (<60 g/dL), and 230 (44.4%) had latent zinc deficiency (60-80 μg/dL).

Zinc deficiency is associated with dermatitis, hair loss, anemia, dysgeusia, growth disturbance, and gonadal dysfunction [15], and replacement therapy is often performed because it is one of the causes of ESA hyporeactive anemia, according to the guidelines [16].

Until 2017, no drugs for the treatment of hypozincemia were covered by insurance, and polaprezinc was often used off-label.

However, there has been an accumulation of hypocopperemia cases during drug administration, and an alert for copper deficiency was issued in 2016. In 2017, zinc acetate, previously indicated for the treatment of Wilson's disease, was extended for hypozincemia. In this case, 50 mg/day of zinc supplementation was used, but copper deficiency was also noted.

In addition, 50-100 mg of zinc acetate contains more zinc (50-100 mg more zinc) compared to 150 mg of polapresinque (34 mg of zinc), and there may be a difference in the risk of hypocopperemia depending on the amount of zinc supplementation. Dr. Okamoto et al. reported [17] that zinc acetate significantly increased zinc but also significantly decreased serum copper compared to polaprezinc, whereas polaprezinc did not significantly decrease serum copper.

According to the 2018 guidelines for the treatment of zinc deficiency [15], the guideline for zinc supplementation is 50-150 mg of zinc per day for adults. However, there are no guidelines for zinc supplementation amounts for dialysis patients.

Since serum copper is less than 10 μg/dL in many cases when copper deficiency develops, the copper deficiency should be noted when serum copper is 20-30 μg/dL and serum zinc is greater than 200 g/dL.

In the present case, the laboratory finding 32 weeks before admission revealed a serum copper level of 21 μg/dL and a serum zinc level of 143 g/dL; therefore, a reduction or discontinuation of the zinc preparation should have been considered.

However, even in the absence of nutritional problems, as in the present case, an increase in zinc supplementation may cause hypocopperemia, suggesting the need for periodic measurements of these trace elements.

## Conclusions

Here, we report a case of myelodysplastic syndrome-like anemia caused by hypocopperemia while consuming zinc acetate.Zinc deficiency is one of the causes of ESA hyporeactive anemia and replacement therapy is often performed in dialysis patients. However, increasing zinc supplementation may cause hypocopperemia. Although there is no clear standard for zinc supplementation in dialysis patients, it is important to check serum copper levels before and during zinc replacement therapy. 
